# Differential expression of microRNAs during fiber development between fuzzless-lintless mutant and its wild-type allotetraploid cotton

**DOI:** 10.1038/s41598-017-00038-6

**Published:** 2017-01-31

**Authors:** Runrun Sun, Chengqi Li, Jinbao Zhang, Fei Li, Liang Ma, Yangguang Tan, Qinglian Wang, Baohong Zhang

**Affiliations:** 1 Henan Collaborative Innovation Center of Modern Biological Breeding, Henan Institute of Sciences and Technology, Xinxiang, Henan 453003 People’s Republic of China; 2 Department of Biology, East Carolina University, Greenville, NC 27858 USA

## Abstract

Cotton is one of the most important textile crops but little is known how microRNAs regulate cotton fiber development. Using a well-studied cotton fiberless mutant Xu-142-fl, we compared 54 miRNAs for their expression between fiberless mutant and its wildtype. In wildtype Xu-142, 26 miRNAs are involved in cotton fiber initiation and 48 miRNAs are related to primary wall synthesis and secondary wall thickening. Thirty three miRNAs showed different expression in fiber initiation between Xu-142 and Xu-142-fl. These miRNAs potentially target 723 protein-coding genes, including transcription factors, such as MYB, ARF, and LRR. ARF18 was newly predicted targets of miR160a, and miR160a was expressed at higher level in −2DPA of Xu-142-fl compared with Xu-142. Furthermore, the result of Gene Ontology-based term classification (GO), EuKaryotic Orthologous Groups (KOG) and Kyoto Encyclopedia of Genes and Genomes (KEGG) analysis shows that miRNA targets were classified to 222 biological processes, 64 cellular component and 42 molecular functions, enriched in 22 KOG groups, and classified into 28 pathways. Together, our study provides evidence for better understanding of miRNA regulatory roles in the process of fiber development, which is helpful to increase fiber yield and improve fiber quality.

## Introduction

Cotton is not only one of the most important fiber-producing crops but also a model species to investigate cell wall formation and cellulose biosynthesis. Cotton fiber development undergoes four overlapping stages: initiation, elongation (primary wall synthesis), secondary wall thickening, and maturation^1^. Fiber cells initiate usually on or prior to the day of anthesis (DPA) and approximately ends at 2 DPA, which impacts the lint numbers from a single cotton seed and then further affect fiber yields^2^. After fiber initiation, single fiber cell undergoes elongation stage at approximately 5–20 DPAs, followed by secondary wall formation at approximately 21–30 DPA, and maturation at approximately 31–50 DPA^3–7^. Cotton fiber cell is the fastest growing and longest single cell in higher plants^4,8^. Therefore, cotton fiber is a perfect model to study plant cell elongation and its regulated mechanisms. However, the molecular mechanisms for controlling cotton fiber is unclear although there are many researches in the past decade.

MicroRNAs (MiRNAs) are a category of endogenous non-coding single-stranded RNAs, which regulate gene expression at the post-transcription levels by degrading corresponding mRNAs or inhibiting mRNA translation^9,10^. As one of the most important gene regulators, miRNAs play multiple roles in the processes of plant growth and development, such as organ development^11^, signal transduction, phase change^12^, and defense against stresses^13–18^.

Over the past decades, miRNAs have been identified in many plant species^19,20^. However, compared with other plants, the investigation of miRNAs in cotton is much beyond other plant species^21–34^, and the majority of those studies focus on miRNA expression in cotton growth and development^35^. However, only few reports focus on miRNA regulatory roles in cotton fiber development^36^. In 2007, three individual miRNAs, including miR414, miR396, miR782, were predicted to target fiber protein Fb23, callous synthase and fiber quinone-oxidoreductase, respectively; these three genes play essential roles in the process of cotton fiber differentiation^34^. Later, one study show that miR162 was highly expressed in immature fibers and ovules^33^. Currently, 34 conserved miRNA families were identified in cotton fiberless mutant Xu-142-fl and its wildtype Xu-142 using deep sequencing technology, and many of these miRNAs are significantly expressed between the two different cotton genotypes^24^. Since then, the research of miRNAs related to cotton fiber development has attracted more and more attention in the cotton miRNA-related research. Liu *et al.* (2014) identified 54 miRNAs including 47 conserved and 7 new miRNAs from island cotton using deep sequencing; their study also show that miR160, miR167, miR171, miR172 and miR827 were highly expressed in fiber initiation stage comparing to the elongation and secondary wall biosynthesis stage^37^. At the same time, several laboratories reported hundreds of miRNAs in cotton ovules and fibers^25,38–40^. Xie *et al.* (2015) identified 65 conserved miRNA families in cotton ovules with initiated fiber and leaves using the first-generation deep sequencing technology; among these miRNAs, the expression of 32 miRNA families were difference between ovule and leaf tissues^41^.

Although those studies have been reported on miRNA expression related to fiber development, no single report has attempted to compare the miRNA expression profiles among multiple fiber development stages and the regulatory mechanism is still unclear. In this study, we studied the expression profiles of 54 miRNAs in cotton ovules, fibers, cotyledons, leaves and flower buds in a well-studied cotton fuzzless-lintless mutant (Xu-142-fl) and its wildtype Xu-142. We selected these 54 miRNAs based on previous reports; these miRNAs either play important role in plant development or are differentially expressed in a certain cotton fiber development stage. Our result shows that the majority of miRNAs were down-regulated in cotton fiber development, suggesting that those tested miRNAs may play positive role in different fiber development stages. 33 miRNAs showed different expression patterns in fiber initiation between Xu-142 and Xu-142-fl. Our study also show that those miRNAs target many transcription factors, suggesting that these miRNA get involved in cotton fiber development potentially through targeting different transcription factors.

## Materials and Methods

### Plant growth condition and material preparation

Upland cotton (*Gossypium hirsutum* L.) cv. fuzzless-lintless mutant Xu-142-fl and its wildtype Xu-142, kindly provided by Cotton Research Institute of the Chinese Academy of Agricultural Sciences, were grown at the experimental field of the Henan Institute of Science and Technology, Xinxiang, Henan Province (China). Flowers were tagged on the day of anthesis (0 DPA), −2 and −1 DPA flowers were estimated by the size of flower bud. The bolls of two genotypes were harvested at −2, −1, 0, 1, 2, 5, 10 and 30 DPA and put on ice immediately. Ovules, including −2, −1, 0, 1, 2 DPA of Xu-142 and all investigated fiber development stages of Xu-142-*fl*, were carefully dissected from each bolls; fiber of Xu-142, including 5, 10 and 30 DPA, was separated from ovule. In addition, 8 days-old cotyledon, the first truly leaf and bud (a diameter of approximately 0.5 to 1 cm) were also harvested. At least three biological replicates were collected for each samples at each developmental stage. All collected samples were frozen in liquid nitrogen immediately and then stored at −80 °C freezer until further use.

### RNA isolation and reverse transcription

Total RNAs were extracted from ovules, fibers, cotyledons, leaves and shoot buds using the Biospin Plant Total RNA Extraction Kit (BioFlux) according to the manufacturer’s instructions. Briefly, tissues were ground into fine powder in liquid nitrogen, and transfer the powder into 1.5 ml centrifuge tube with lysis and PLANTaid, then immediately vortex several times to mix well and stand at room temperature for 5 minutes. After adding wash buffer, 40 μL DNase Buffer was added to the spin column to purify RNA. The quantity and purity of RNAs were measured by NanoDrop ND-2000 Spectrophotometer (Thermo Scientific, ND-2000, USA).

Based on previous studies, a total of 54 miRNAs were selected for this study; these miRNAs are either important to plant development or differential expressed at a certain development stage of cotton differentiation and development. A total of 200 ng total RNAs extracted from difference tissues was used to transcribe into cDNA using TaqMan MicroRNA Reverse Transcription Kit (Applied Biosystems, Foster City, CA, USA) as our previous reports^42,43^. MiRNA-specific stem loop primer was employed to run reverse transcription. According to the kit supplier’s protocol, 15 μL total reverse transcription reaction mixture contains 1.5 μL Reverse Transcription buffer, 1.0 μL MultiScribeReverse Transcriptase, 0.15 μL dNTPs, 0.19 μL RNase inhibitor, and 200 ng total RNAs. When the reaction was done, 80 μL DNase free water was added to dilute the RT-PCR products, and vortex gently to mix well, then store at −20 °C for further qRT-PCR.

### Quantitative real-time PCR

The real-time RT-PCR was performed with an Thermo Scientific PikoRea Real-Time PCR System (Thermo Scientific, PikoReal Real 96, USA). According to our reported previously, each reaction includes 1 μL cDNA, 2 μL forward and reverse primers, 5 μL 2× SYBR green mixture and 2 μL DNase free water. The qRT-PCR was performed as following: 10 min at 95 °C, followed by 40 cycles of 15 s at 95 °C and 1 min at 60 °C. Based on our previous study, UBQ14 is the most reliable reference gene in cotton; thus we used it as reference gene to normalize the tested gene expression value. SPSS was employed to analyze the biostatistics significance. A heat map was generated with MeV (Multi Experiment Viewer) according to previous reported^42,43^.

### Predication of miRNA targets and function analysis

The mature miRNA sequences and the CDS sequences that extracted from CottonGen database (http://www.cottongen.org) were submitted to *psRNATarget* database (http://plantgrn.noble.org/psRNATarget/) to search miRNA targets. The optimized criteria as follows: the hspize value in the range from 1 to 17 nt; the central mismatch between 10 and 11 nt; the rest parameters were defined by the program.

Identified miRNA targets were performed alignment against KEGG Orthology (KO) and EuKaryotic Orthologous Groups (KOG) database for Kyoto Encyclopedia of Genes and Genomes (KEGG) pathway enrichment and KOG term classification. To visualize the biological process, cellular component and molecular function that miRNA targets involved in, the nr database and Blast2go software were employed to analyze miRNA targets Gene Ontology (GO) term classification.

## Results

### MiRNA expression pattern in wildtype Xu-142

**Figure 1:**
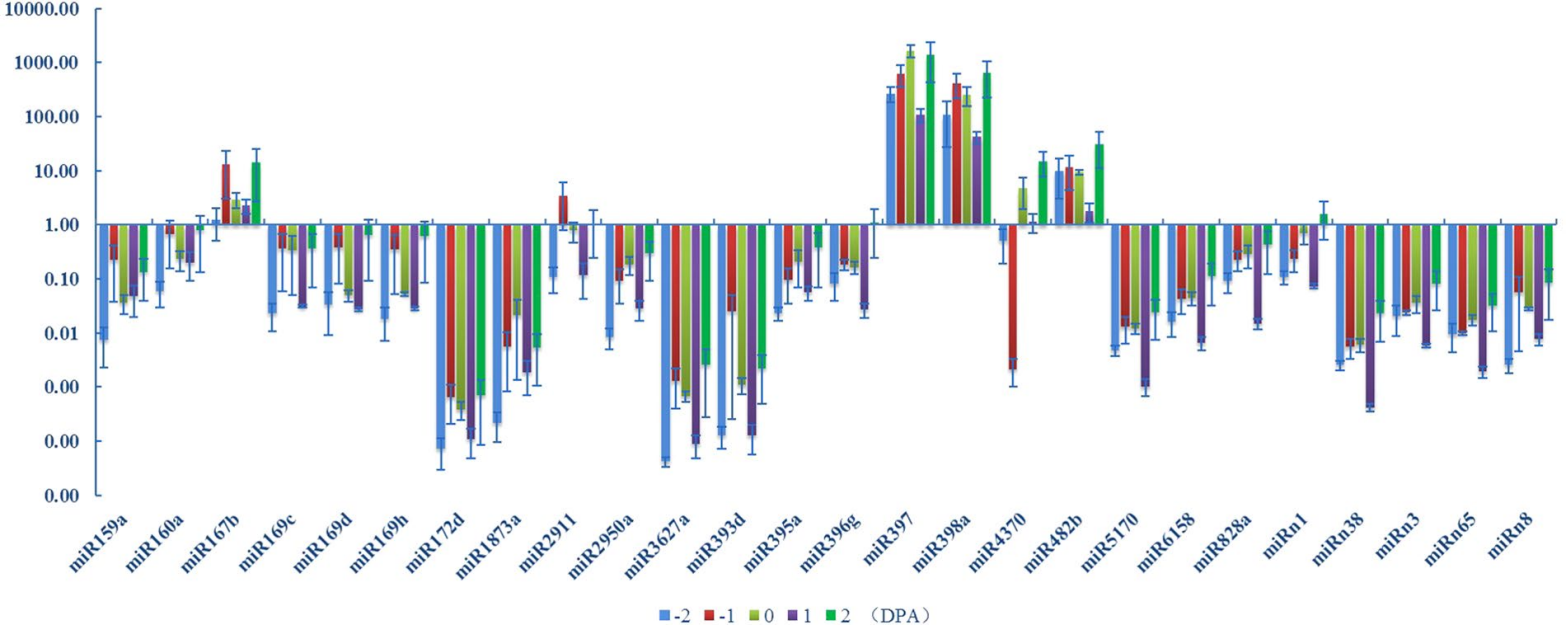
A total of 54 miRNAs were selected and profiled during the cotton fiber initiation and early development. This figure only show the miRNAs with significant expression during fiber initiation in cotton cultivar *Xu-142*.

To study the miRNA expression profiles in wildtype Xu-142, quantitative real time PCR (qRT-PCR) was performed for 54 miRNA in different tissues, including cotyledons, leaves, buds, ovules and fibers. As shown in Fig. 1, certain miRNAs show similar expression patterns in five different fiber development stages while other miRNAs were differentially expressed in immature ovules and fiber bearing ovules, which might indicate miRNAs functional divergence during fiber development periods. For example, four miRNAs, miR167b, miR397, miR398a and miR482b, were high expressed in fiber initiation stage (Fig. 1). Sixteen out of 54 miRNAs (approximately 29.6%), including miR159a, 160a, 167b, 169c, 169d, 169h, 172d, 1873a, 2950a, 3627a, 395a, 397, 398a, 4370, n1 and n8, were expressed significantly lower in the ovules of −2DPA than that in the ovules of 0DPA. However, nly miR4370 show significant lower expression in the ovules of −1DPA than that in the ovules of 0DPA (Fig. 1). Additionally, 14 miRNAs, including miR2911, 3627a, 393d, 396g, 397, 398a, 482b, 5170, 6158, 828a, n1, n3, n38 and n65, show similar expression profiles during cotton fiber early development (Fig. 1).

**Figure 2:**
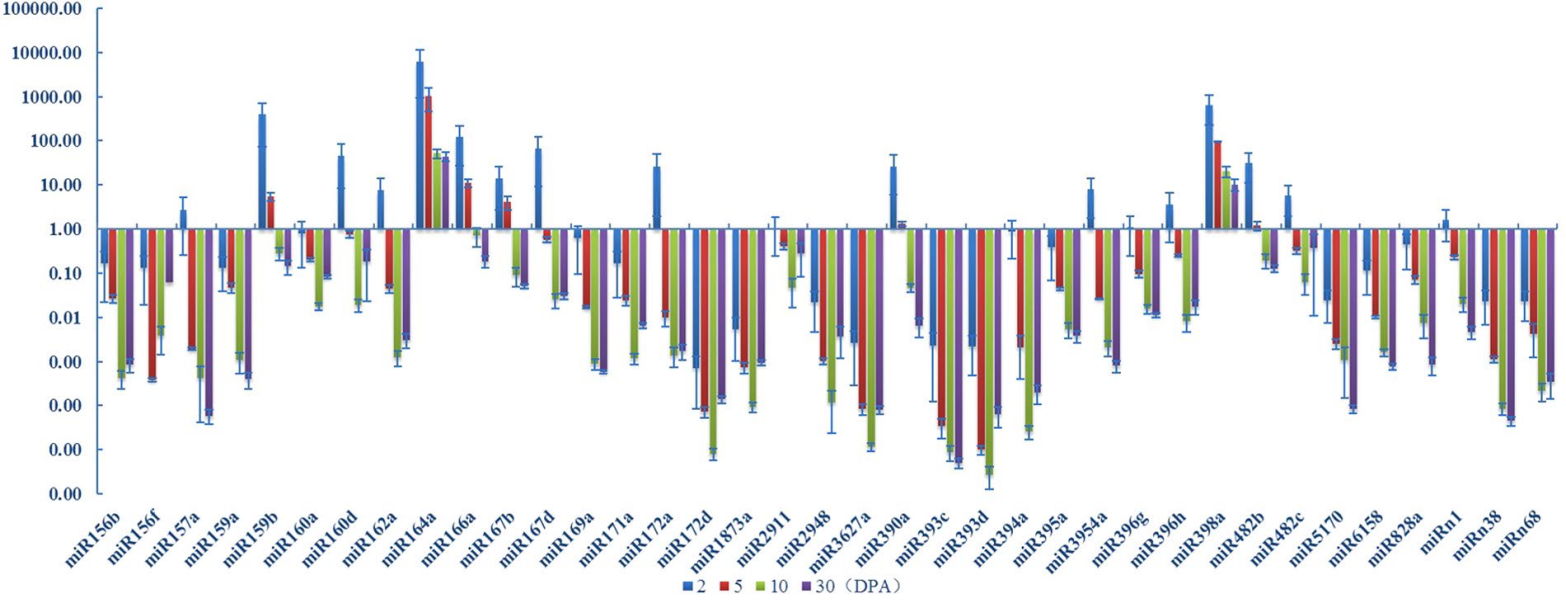
A total of 54 miRNAs were selected and profiled during the cotton fiber initiation and early development. This figure only show the miRNAs with significant expression during fiber elongation and secondary wall formation in cotton cultivar *Xu142*.

Many miRNAs show exclusively low expression in specific tissues. For example, 17 out of 54 miRNAs display especially lower expression in the 10 DPA fiber whereas show higher expression in the ovules of 2 DPA, suggesting that those miRNAs may have different role in fiber rapid elongation (Fig. 2). In addition, a similar expression profile was also found in 19 miRNAs, including miR157a, 159a/b,164a, 166a, 167b, 169a, 390a, 393c, 395a, 3954a, 396g, 398a, 482b, 5170, 6158, 828a and n1/38; all these miRNAs were expressed at a lower level in 30 DPA fiber than that in other developmental stages (Fig. 2). The expression of miR156f was unique, and show higher expression in 2 DPA, medium levels in 10 and 30 DPA, and lower expression in 5 DPA (Fig. 2).

### MiRNA expression pattern in fiber initiation in fiberless mutant and its wildtype

**Figure 3:**
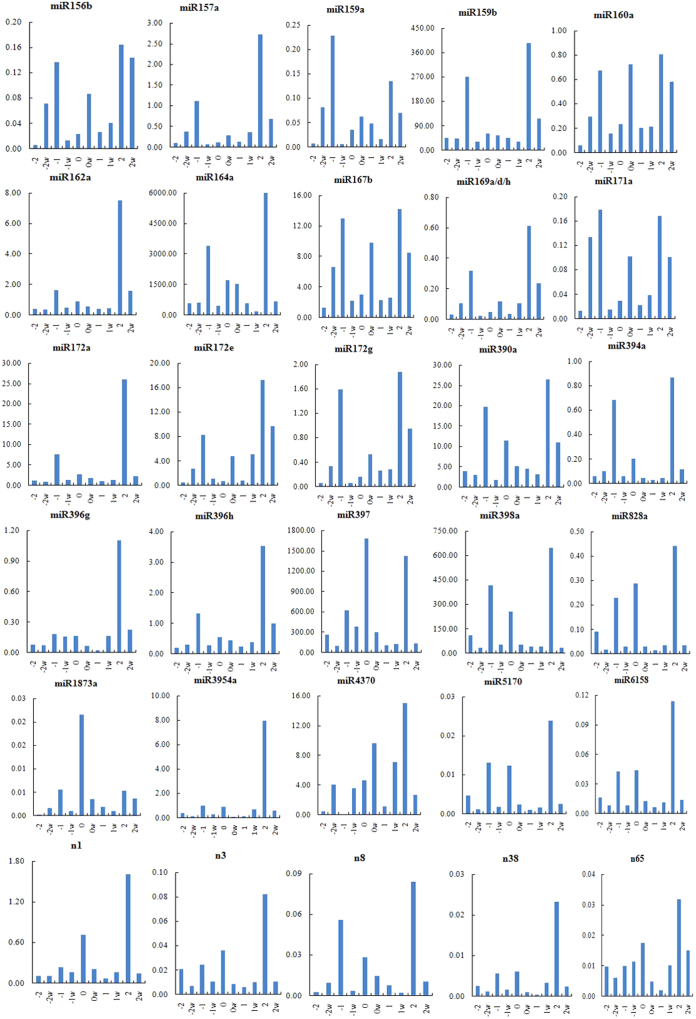
Among 54 tested miRNAs, many are differentially expressed between the cotton fiberless mutant Xu-142-fl (marked as w in the figure) and its wildtype Xu-142. In this figure, only 30 miRNAs are presented with different development stages, which show significant expression between cotton fiberless mutant Xu-142-fl and its wildtype Xu-142.

At −2 DPA, miR156b, 157a, 159a, 160a, 167b, 169a/d/h, 171a, 172e/g, 1873a, and 4370, display significant higher expression in Xu-142-fl than that in Xu-142, whereas miR828a exhibit relatively low expression in Xu-142fl compared with Xu-142. At 0 DPA, some miRNAs show similar expression profile. For example, the expression levels of miR3954a, 397, 398a, 5170, 828a, n3 and n38 were significant lower in Xu-142-fl than that in the wildtype Xu-142. Similarly, miR397, 398a, 4370, 5170, 6158, 828a, n1, n3, n8 and n38 show significantly higher expression at 2 DPA in the wildtype ovule than that in the fiberless mutant Xu-142-fl. (Fig. 3). Additionally, seven miRNAs, miR159b, 164a, 167b, 172a, 390a, 397 and 398a, show similar expression profiles during fiber initiation in both Xu-142 and Xu-142-fl (Fig. 3), which suggests those miRNAs play important roles in fiber initiation stage.

### Potential functions of miRNA in cotton fiber development

**Figure 4:**
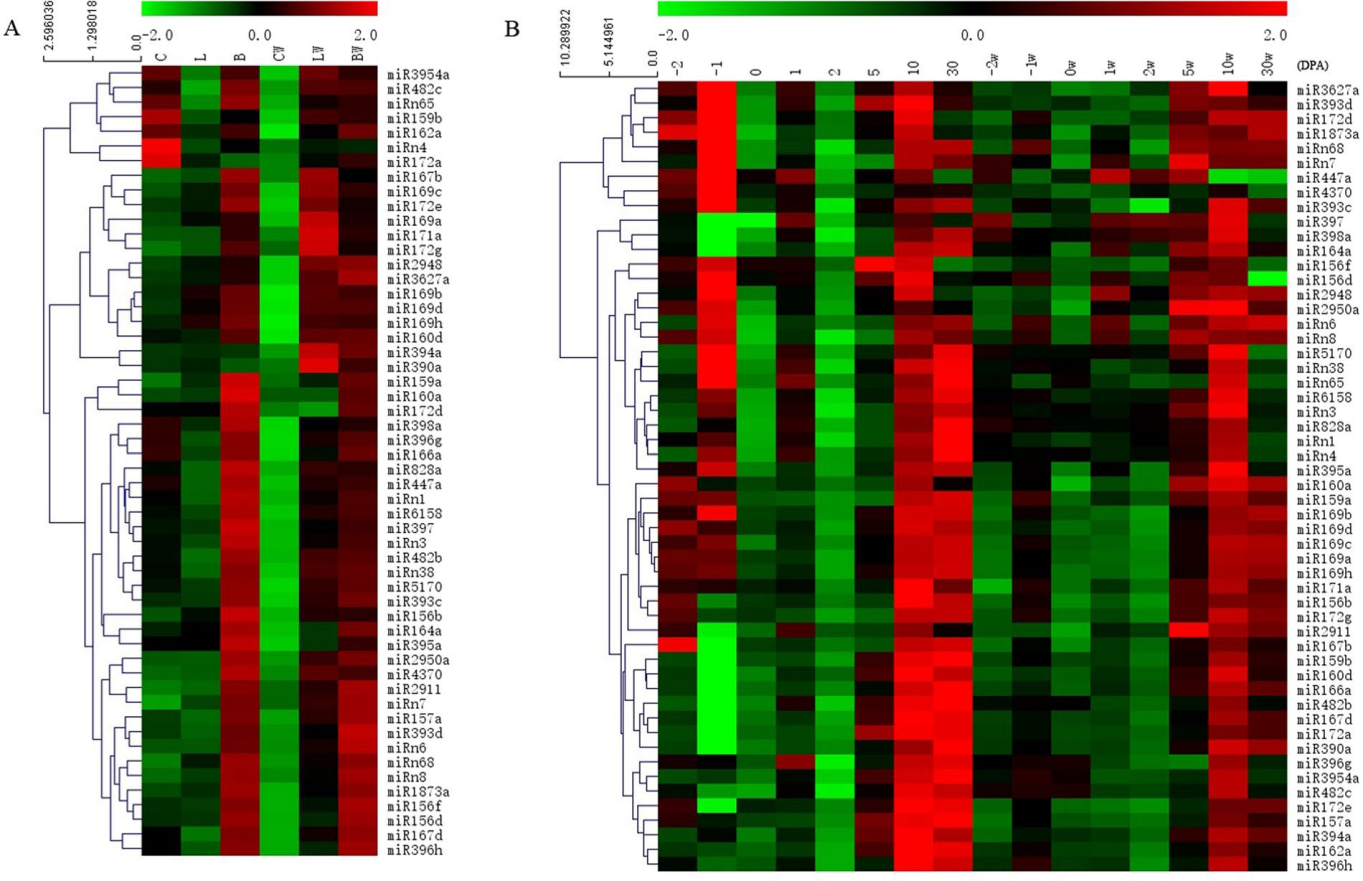
Heatmap of miRNAs expression profile in all investigated tissues. The ranking of expression was showed using different colors: higher expression was designed red; median expression black, reduced expression green; (**A**) miRNAs expression profile in non-fiber tissues; (**B**) The expression of tested miRNAs in fiber development stage of fiberless mutants and its wildtype.

To further characterize miRNA expression patterns, we employed Mev to construct miRNA expression profiles in cotton fibers and non-fiber tissues, respectively (Fig. 4A). The tested miRNAs exhibit different expression patterns during vegetable organs and reproductive tissues, and clustered into different groups with similar profile. Among three non-fiber organs of the fiberless mutant and its wildtype, most miRNAs show lower expression in cotyledons whereas exhibit higher expression in flower buds, this might indicate that all the tested miRNAs play different role in the reproductive stage (Fig. 4A). It is worth to note that the expression of most miRNAs was higher in Xu-142-fl leaves but exhibit lower expression in the leaves of Xu-142, which suggests that those miRNAs might play different role in different genotypes (Fig. 4A). In addition, 11 miRNAs, including miR3954a, 482c, n65, 159b, 162a, 172a, 398a, 396g, 166a, 447a and n4, also show higher expression in cotyledon of Xu-142, indicating that those miRNAs might play different role in the early stage of cotton development (Fig. 4A).

As shown in Fig. 4B, the majority of miRNAs were expressed most at the 10 and 30 DPA in both Xu-142 and Xu-142-fl, implying that those miRNAs may regulate fiber elongation development. Eleven miRNAs, 156b/f, 159a, 160a, 167b, 169a/b/c/d/h, 172d/e/g, 1873a, 2950a, 3627a, 4370 and n8, were higher expression in −2DPA of Xu-142 but were lower expression in −2DPA of Xu-142-fl, suggesting that those miRNAs play negative role in the fiber initiation stage. Eighteen out of 54 miRNAs (approximately 33.3%), including miR156b, 159a, 160a, 164a, 172g, 1873a, 2911, 2950a, 3627a, 397, 398a, 447a, 5170, n3, n6, n7, n68, and n8 show lower expression in 5DPA ovules of Xu-142, whereas exhibit higher expression in 5DPA ovules of Xu-142-fl. Thus, these miRNAs might serve as regulators during fiber elongation development. In contrast, the expression of miR447a was lower in 10 DPA fiber of Xu-142-fl but expressed higher at the same stage of Xu-142.

### MiRNA target identification

The *Gossypium hirsutum* genome sequence have been reported previously^44,45^, and deposited in the CottonGen database. The transcript sequences were used for miRNA target prediction. After remove redundancy sequences, 723 genes were predicted to be miRNA targets. Those genes were further performed KOG, KEGG and GO search against to KOG, KO and nr database to predict the miRNA target function (Table 1).

**Table 1:** MiRNA targets annotation in database.

	Number	Percent (%)
KOG	254	35.13%
NR	723	100%
KEGG	254	35.13%
GO	299	41.36%
Total	723	100%

### Functional classification of miRNA targets

**Figure 5:**
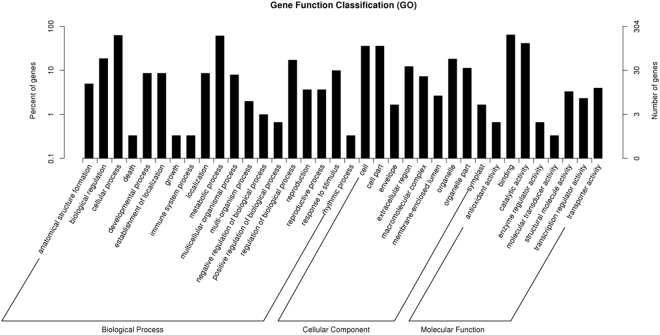
The GO classification of miRNA targets. These miRNA targets are predicted by computational program described in the method section. The majority of miRNA targets have been confirmed experimentally in previous reports.

Gene Ontology is a tool to predict gene function. In this study, a total of 222 biological processes, 64 cellular components and 42 molecular functions were categorized in Gene Ontology classification analysis. The top three biological processes are Cellular process (GO:0009987), metabolic process (GO:0008152), biological regulation (GO:0065007). Similarly, the most frequent cellular component where most miRNA targets are located in cell (GO:0005623), followed by cell part (GO:0044464) and organelle (GO:0043226). Additional, The majority of miRNA targets have the molecular function of binding (GO:0005488), catalytic activity (GO:0003824) and transporter activity (GO:0005215) (Fig. 5).

**Figure 6:**
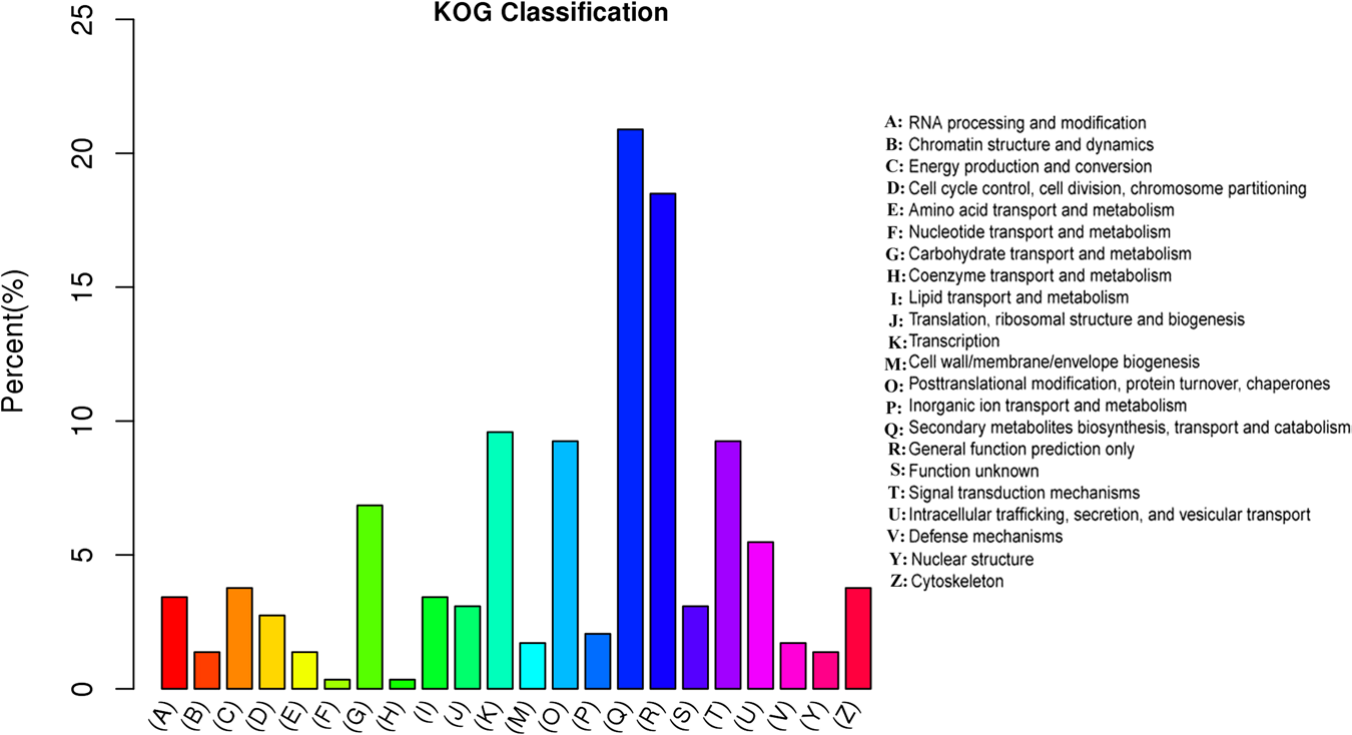
The KOG classification of miRNA targets. These miRNA targets are predicted by computational program described in the method section. The majority of miRNA targets have been confirmed experimentally in previous reports.

A total of 254 miRNA targets were assigned to 22 EuKaryotic Orthologous Groups (KOG) functional categories. Among these, 61 out of 331 genes were attributed to secondary metabolites biosynthesis, transport and catabolism; 54 genes (approximately 16.3%) were mapped to general function only; one gene was classified into nucleotide transport, metabolism and coenzyme transport and metabolism, respectively (Fig. 6).

**Figure 7:**
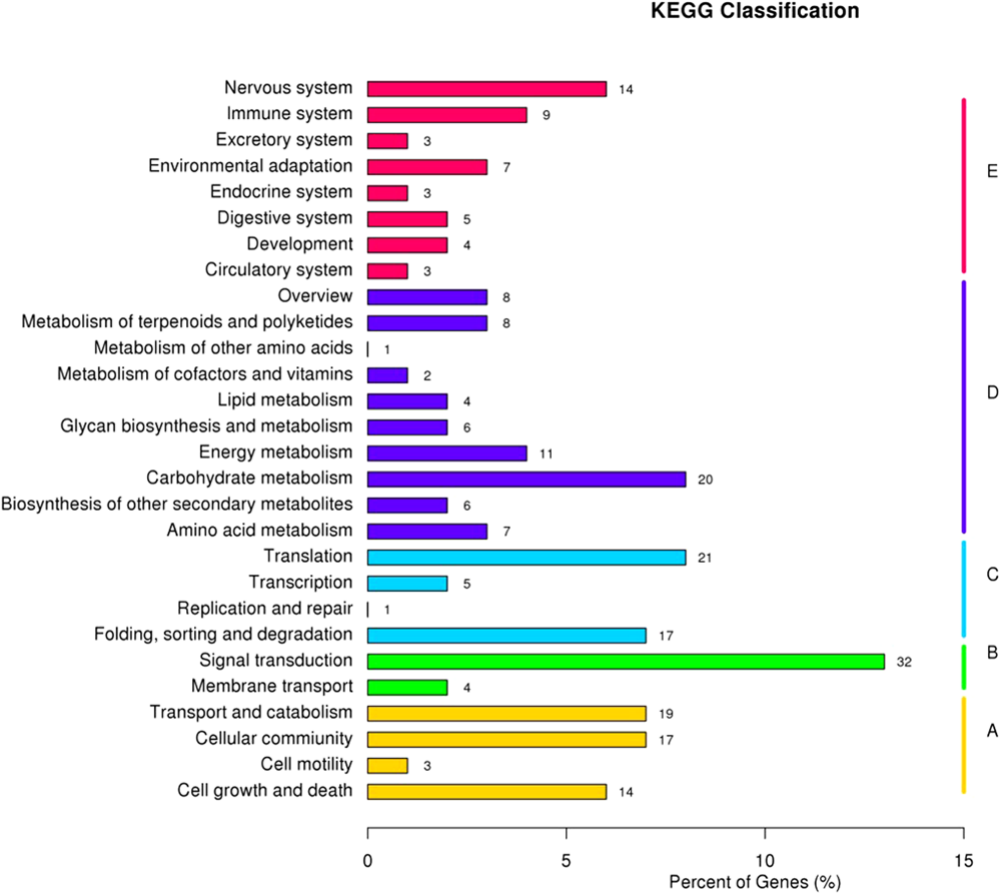
The KEGG classification of miRNA targets. These miRNA targets are predicted by computational program described in the method section. The majority of miRNA targets have been confirmed experimentally in previous reports. These classification includes cellular processes (**A**), environmental information processing (**B**), genetic information processing (**C**), metabolism (**D**) and organismal system (**E**).

Kyoto Encyclopedia of Genes and Genomes (KEGG) analysis classified 254 miRNA targets into 28 pathways, including 4 Cellular Processes (A), 2 Environmental Information Processing (B), 4 Genetic Information Processing (C), 10 Metabolism (D) and 8 Organismal Systems (E). A total of 32 (8.99%) sequences were mapped to pathway in signal transduction, whereas translation was represented by 21 (5.9%) sequences; 5 were shown to be involved in transcription; 8 genes were related to metabolism of terpenoids and polyketides and overview, respectively (Fig. 7).

## Discussion

Since the first miRNA was reported in plants in 2002, lots of miRNAs and corresponding targets have been reported in many plant species including some important crops. Cotton, as one of the most important natural fiber product species, provides income for about 100 million families among approximately 150 countries globally. In the past decades, several studies have been reported to inquiry the miRNA expression during cotton fiber differentiation and development^37,38,40,41,46–51^. However, the majority of these studies were targeted in one or a few developmental stages, no one study has been focus on the different stages cross cotton fiber initiation and development.

It is well-known that cotton fiber development shares many similarities with Arabidopsis leaves trichomes in cellular and genetic features^52^. In Arabidopsis, miR156, which targets Squamosa promoter binding protein-like (SPL) transcription factors, temporally controls phase change and trichome distribution^53^. In this study, we found that miR156b was expressed at a higher level in the −2 DPA ovule of Xu-142-fl than that in its wildtype Xu-142 (Fig. 3), indicating that the target gene, SPL, plays promoting role in early fiber initiation stage. It has been reported that miR156 and miR172 regulates the phase transition in complementary patterns^54–57^. Interestingly, miR172a shows relatively higher expression pattern in the −2 DPA ovule of Xu-142 than that in Xu-142-fl (Fig. 3), which suggests that the similar regulation patterns in fiber and flowers.

MYB transcription factor plays a role in trichome development in cotton leaves and fiber development^58–60^. As previous reported, cotton GhMYB109 and GaMYB2 are homolog of GL1, which mediates fiber development^61^. In this study, several MYB family members were predicted to be the targets of miR159b and miR828; the expression of miR159b and miR828a was lower in fiberless mutant Xu-142-fl than that in the wildtype Xu-142 in the developmental stages of 0, −1 and −2DPA, respectively (Fig. 3).

Auxin, as a critical plant hormones, regulats seed and fiber development. Both miR160 and miR167 may regulate cotton fiber development via targeting the auxin response factors (ARF). MiR160 targets ARF10, ARF16 and ARF17 whereas ARF8 and ARF6 was identified as the targets of miR167^62,63^. In our study, ARF18 was newly predicted the target of miR160a, and miR160a was expressed at a higher level at −2DPA of Xu-142-fl compared with Xu-142 (Fig. 3), indicating that ARF18 plays positive role in the development of fiber initiation. Similarly, ARF17 was also the positive regulator in cotton fiber early development.

miR164a may target NAC transcription factors. The expression of miR164a was higher in immature ovules of Xu-142 than that in Xu-142-fl (Fig. 3), suggesting that NAC might act as a native regulator in cotton fiber early development. This result was consistent with a previous report^26^. In addition, HD-ZIP III was cleaved by miR165/166, and show higher expression in fibers^26^. HD-ZIP III was also the target of miR166a in this study, and the expression of miR166a was reduced as the cotton fiber development (Fig. 2), implying that HD-ZIP III was promoting cotton fiber secondary wall formation.

LRR was targeted by miR390a, and the expression of miR390a was higher at 2 to 5 DPA and sharply declined at 10DPA, implying that miR390a regulated LRR might play positive role in initiating rapid fiber elongation stage (Fig. 2). This result is consistent with a previous report^41^. The putative target gene of miR397, Laccase, regulates copper homeostasis and lignin biosynthesis^64,65^. Several members of LAC was predicted to be the targets of miR397. The expression of miR397 was higher in most fiber development stages particular in fiber elongation stage and secondary wall formation, resulting in the down-regulation of the laccase gene (Fig. 4). We speculate that, less laccase may reduce lignin production, causing the epidermal cell wall loosening. This reaction prompts fiber bulging and initiation.

MiRNA mediated network in ovule and fiber development in cotton fiberless mutants and its wioldtype is very complicated. By using qRT-PCR, we examined the expression patterns of 54 miRNAs which has been reported to involve in fiber development. We also provided the GO-based classification and KEGG pathway enrichment of tested miRNAs. This study provides a comprehensive comparison of 54 miRNA expression profiles during multiple developmental stages of cotton fiber initiation and development, which may provide the potential regulatory roles in cotton fiber development.
